# Reversibility of apoptosis in cancer cells

**DOI:** 10.1038/sj.bjc.6604802

**Published:** 2008-12-16

**Authors:** H L Tang, K L Yuen, H M Tang, M C Fung

**Affiliations:** 1 Department of Biology, The Chinese University of Hong Kong, Shatin, Hong Kong SAR, China; 2 Department of Biology, Iowa State University of Science and Technology, Ames, IA 50011, USA

**Keywords:** apoptosis, death, reversibility, survive, tumour

## Abstract

Apoptosis is a cell suicide programme characterised by unique cellular events such as mitochondrial fragmentation and dysfunction, nuclear condensation, cytoplasmic shrinkage and activation of apoptotic protease caspases, and these serve as the noticeable apoptotic markers for the commitment of cell demise. Here, we show that, however, the characterised apoptotic dying cancer cells can regain their normal morphology and proliferate after removal of apoptotic inducers. In addition, we demonstrate that reversibility of apoptosis occurs in various cancer cell lines, and in different apoptotic stimuli. Our findings show that cancer cells can survive after initiation of apoptosis, thereby revealing an unexpected potential escape mechanism of cancer cells from chemotherapy.

Chemotherapy is one of the major cancer treatments by promoting cancer cells into different types of cell death including apoptosis (Johnstone *et al*, 2002; Perona and Sánchez-Pérez, 2004; Brown and Attardi, 2005; Li *et al*, 2008). Accumulating studies reported that cancers initially retreated in response to chemotherapy, but returned during repeated courses of treatment (Norton and Simon, 1977; Stephens and Peacock, 1977; Davis and Tannock, 2000; Wu and Tannock, 2003; Kim and Tannock, 2005). Although the mechanisms of the cancer recurrence are not well understood, it is generally believed that repopulation of surviving cancer cells during the intervals between treatments is an important cause of the treatment failure (Kim and Tannock, 2005). The survival of cancer cells during treatments has been mainly attributed into the deficiency of apoptotic pathways in cancer cells (Letai, 2008), anticancer drug resistance of tumorigenic stem cells (Dean *et al*, 2005), and inefficiency of drug penetration into solid tumours for achieving a therapeutic effect (Minchinton and Tannock, 2006). In this study, we found that cancer cells could survive even after initiation of apoptosis, and this was observed in various cancer cell lines and in different apoptotic stimuli. Our findings reveal another possibility that may contribute into the cancer cell survival during therapy, reversibility of apoptosis in cancer cells.

## Materials and methods

### Cell culture

Human cervical cancer HeLa cells, skin cancer A375, liver cancer HepG2, breast cancer MCF7 cells (from American Type Culture Collection) were cultured in DMEM (Dulbecco's minimum essential medium) supplemented with 10% heat-inactivated FBS (fetal bovine serum), 100 U ml^−1^ penicillin and 100 *μ*g ml^−1^ streptomycin (Gibco, Carlsbad, CA, USA), at 37°C under an atmosphere of 5% CO_2_/95% air. Human prostate cancer PC3 cells were cultured in the same condition with RPMI-1640 medium. Cells were seeded on tissue culture plates until the cell density reached 70% confluency before being subjected to each experiment. Apoptotic stimuli jasplakinolide (Invitrogen, Carlsbad, CA, USA), staurosporine (Sigma, St Louis, MO, USA) and ethanol (Scharlau, Barcelona, Spain) were applied to the cells.

### Living cell staining

Cells were grown to 70% confluence on a glass coverslip (Marienfeld, Lauda-Künigahofen, Germany). Mitochondria and nucleuses were stained with 50 nM MitoTracker Red CMXRos (Invitrogen) and 250 ng ml^−1^ Hoechst 33342 (Invitrogen), respectively for 20 min, and the cells were washed two times with PBS and then cultured in suitable fresh medium (Invitrogen).

### Real-time living cell microscopy

Cells were cultured in CO_2_-independent medium (Invitrogen) on a thermo-cell culture FCS2 chamber (Bioptechs, Butler, PA, USA) mounted onto the adapter in the stage of an inverted fluorescence microscope Cell Observer (Carl Zeiss, Jena, Germany). Cell morphology was visualised by differential interference contrast (DIC) microscope device, and the mitochondria and nucleuses were by fluorescence with excitation 561 and 405 nm, respectively. Drugs and culture medium were introduced to the cell culture chamber through the perfusion tubes (Bioptechs) connected to the cell chamber. Cell images were captured with a monochromatic CoolSNAP FX camera (Roper Scientific, Pleasanton, CA, USA) using a × 63 numerical aperture (NA) 1.4 Plan-Apochromat objective (Carl Zeiss), and analysed by using AxioVision 4.2 software (Carl Zeiss).

### Confocal microscopy

Confocal cell images were captured with an inverted laser-scanning microscope LSM 5 LIVE (Carl Zeiss), with 1 *μ*m interval between each focal plane. The images were analysed by using LSM image examiner software (Carl Zeiss).

### Biochemical and cell proliferation assays

One thousand cells were grown in a 96-well plate for 24 h, and then treated with different conditions. At each designed time point, cells were subjected to the corresponding assays according to the manufacturer's instructions. The activity of effector caspases was measured by using the homogeneous caspase assay kit (Roche, Mannheim, Germany). The activity of mitochondria was measured by 3-(4,5-dimethylthiazol-2-yl)-2,5 -diphenyltetrazolium bromide (MTT) assay (Sigma). The cell survival was detected by the cell proliferation ELISA BrdU assay kit (Roche). Results of assays were measured by SpectraMax 250 microplate reader (Molecular Devices Corp, Concord, ON, Canada).

### Cell counting

The morphology of the cells was observed by DIC, whereas mitochondria and nucleuses were visualised by fluorescence microscopy. At least 100 cells were examined in at least three independent cell counting.

For cell proliferation, at each indicated time point, the cells were harvested by trypsinisation and thoroughly resuspended. The cells were stained with trypan-blue and counted in triplicate under a microscope with hemocytometer. Total cell number is calculated by multiplying the determined cell density with the total volume of suspension.

### Western blot analysis

Approximately 3 *μ*g protein per lane was separated on a 10% SDS-PAGE gel and transferred onto a Hybond ECL® membrane (Amersham Biosciences, Chalfont St Giles, UK). After blocking, the membrane was incubated overnight at 4°C with 1 : 1000 anti-caspase-3 antibody (Cell Signaling, Danvers, MA, USA) followed by another hour of incubation in the corresponding horseradish peroxidase-conjugated secondary antibody (Bio-Rad, Hercules, CA, USA) at 1 : 5000 dilutions, and the signal was detected with the ECL western blotting detection system (Amersham Biosciences).

## Results

### Survival of HeLa cells from jasplakinolide-induced apoptosis

**Figure 1:**
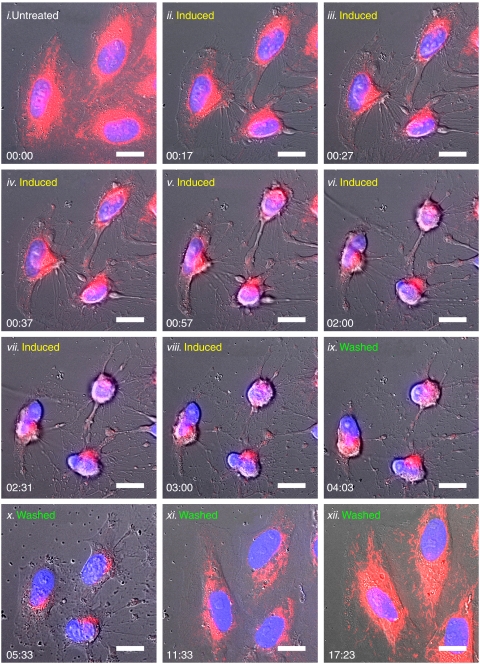
Time-lapse living cell microscopy of HeLa cells under and after jasplakinolide induction. Real-time imaging of the same cells before 0.5 *μ*M jasplakinolide induction (Untreated, *i*), induced for 3 h (Induced, *ii*–*viii*), and then washed and further incubated with fresh culture medium for another 14.5 h (Washed, *ix*–*xii*). Merged images: mitochondria (red) and nucleuses (blue) were visualised by fluorescence, and cell morphology was by DIC. Time presented as hr:min. Scale bar: 10 *μ*m. A corresponding movie is available as the Supplementary Information (Video 1).

**Figure 2:**
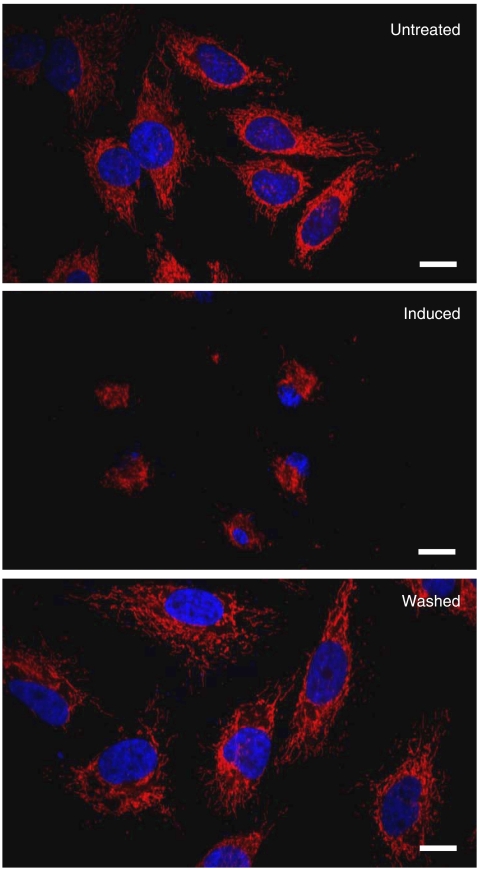
Confocal microscopy for verification on the morphological alternation of HeLa cells during reversibility of apoptosis. Confocal imaging of the HeLa cells before 0.5 *μ*M jasplakinolide induction (Untreated), induced for 3 h (Induced), and the induced cells washed and further incubated with fresh culture medium for another 24 h (Washed). Merged images: mitochondria (red) and nucleuses (blue) were visualised by fluorescence. Scale bar: 10 *μ*m.

**Figure 3:**
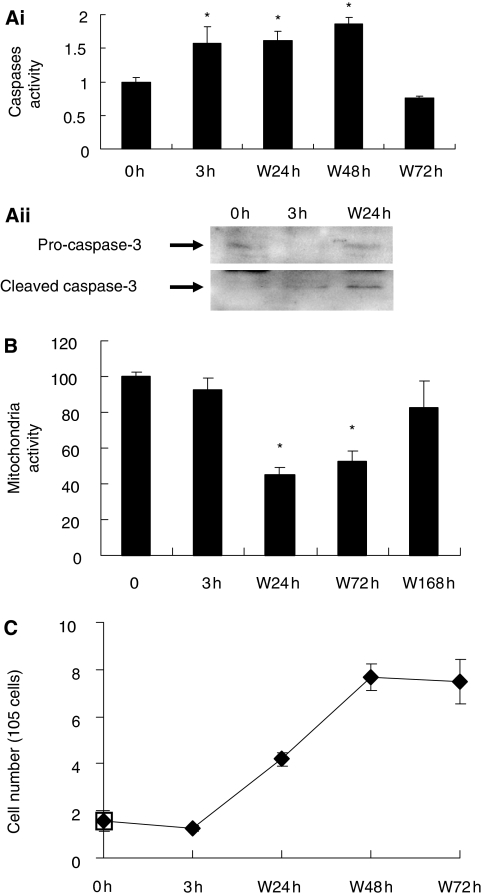
Caspases and mitochondria activity of HeLa cells in different apoptotic stimuli. (**A**) (**i**) Caspases activity of the HeLa cells before the 0.5 *μ*M jasplakinolide induction (0 h), induced for 3 h (3 h), and then washed and cultured for 24 h (W24 h), 48 h (W48 h) and 72 h (W72 h). A thousand of corresponding cells were subjected into caspases assay. The degree of corresponding caspases activities were normalised with the control. (**ii**) Western blot analysis of pro-caspase-3 and cleaved caspase-3 in the total cell lysate from the cells at 0, 3 and W24 h. (**B**) Mitochondria activity of the untreated HeLa cells before the 0.5 *μ*M jasplakinolide induction (0 h), induced for 3 h (3 h), and then washed and cultured for 24 h (W24 h), 72 h (W48 h) and 168 h (W168 h). (Mean±s.d.; ^*^P<0.02; *n*=3). (**C**) Proliferation of the jasplakinolide-induced cells after removal of inducer. HeLa cells were induced with 0.5 *μ*M jasplakinolide for 3 h (3 h), then washed and further incubated in fresh medium for 24 h (W24 h), 48 h (W48 h) and 72 h (W72 h).

Our aim was to induce cancer cells undergoing apoptosis, and investigate whether the apoptotic dying cancer cells could survive after the removal of apoptotic reagents. We initially exposed human cervical carcinoma HeLa cells to an apoptotic inducer jasplakinolide (Odaka *et al*, 2000), and observed the change in the cellular morphology by real-time living cell microscopy. Figures 1 and 2 show that, in untreated healthy cells, mitochondrial network was extensively interconnected and appeared filamentous extended throughout cytoplasm, and the nucleus is in round shape, as previous studies described (Kerr *et al*, 1972; Jacobson *et al*, 1997; Taylor *et al*, 2008). In a 3 h-induction, as expected, the cells displayed morphological landmarks of apoptosis-associated mitochondrial fragmentation, perinuclear redistribution of mitochondria, nuclear condensation and cytoplasmic shrinkage (Figures 1 and 2). The initiation of apoptosis was further confirmed by the biochemical landmarks of apoptosis (Wang, 2001), the activation of apoptotic protease caspases detected by colorimetric caspases assay (Figure 3Ai), the cleavage of pro-caspase-3 (Figure 3Aii) and also the dysfunction of mitochondria by MTT assay (Figure 3B). Then, the apoptotic dying cells were washed and further cultured in a fresh cell medium (the wash). It has been generally assumed that cells displaying all these apoptotic landmarks are determined to die (Kerr *et al*, 1972; Jacobson *et al*, 1997; Green and Kroemer, 2004; Taylor *et al*, 2008). Mitochondrial destruction and activation of caspases have been known to indicate irreversible cell death (Green and Kroemer, 2004; Riedl and Shi, 2004). Here, we reasoned that, if the apoptotic dying cells could survive even after the activation of caspase-3, the cells should be able to restore themselves and proliferate after the wash.

**Figure 4:**
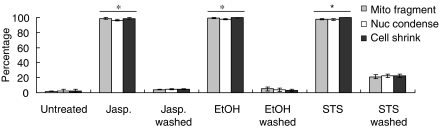
Reversibility of apoptosis in HeLa cells in different apoptotic inductions. Percentage of cells showing mitochondrial fragmentation (Mito. Fragment), nuclear condensation (Nuc. Condense), and cell shrinkage (Cell Shrink) of the control HeLa cells (Untreated), the cell treated with 0.5 *μ*M jasplakinolide for 3 h (Jasp.), 6% (v/v) ethanol for 30 min (EtOH) or staurosporine for 1 h (STS), and the corresponding cells washed and cultured with fresh medium for 24 h (Washed). (Mean±s.d.; ^*^P<0.02; *n*=3).

**Figure 5:**
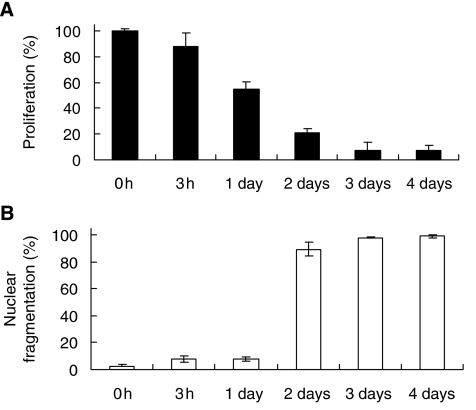
Reversibility of apoptosis after different duration of jasplakinolide-induction. HeLa cells were treated with 0.5 *μ*M of jasplakinolide for 0, 3 h, 1 day, 2 days, 3 days and 4 days. (**A**) Percentage of HeLa cell proliferation (detected by BrdU assay) after removal of the inducer at the indicated time and cultured for another 5 days in fresh medium. (**B**) Percentage of corresponding cells showing nuclear fragmentation (detected by fluorescence microscopy followed by cell counting) at the inducted time before the removal of the inducer.

Interestingly, our results indicate that HeLa cells could actually survive after the apoptotic induction. Our living cell microscopy showed that the shrunk cells regained their normal cellular morphology in 14.5 h after the wash (Figure 1, and Supplementary Video 1). Our confocal microscopy was performed to verify the morphological recovery of the cells in 24 h after removal of the inducer (Figure 2). The cell counting results showed that 96% of the cells displayed all the morphological landmarks of apoptosis in a 3 h-induction, whereas 92% of the cells regained their normal morphology after the wash in 24 h (Figure 4). Besides, the caspases and mitochondria activity also returned back to the level of the control cells in 72 and 168 h after the wash, respectively (Figure 3A and B). Furthermore, survival of the cells after the wash was proven by cell proliferation. The result of cell count showed the increase in the cell number after the removal of the inducer (Figure 3C). The cell survival after reversal was further assessed by the uptake of a DNA synthesis marker bromodexyuridine (BrdU) (Figure 5A). The percentage of proliferation of the cells did not significantly differ between the control and the 3 h-induced cells after the wash (Figure 5A). Our data shows that the morphological and biochemical landmarks of apoptosis vanished in 24 h after the removal of the apoptotic jasplakinolide induction, and the induced cells survived afterward. Noticeably, our time-course study in cell count showed that the percentage of cells displaying nuclear fragmentation increased along the continuous apoptotic induction, and that was inversely proportional to the cell proliferation ability after removal of the apoptotic reagent at that time point (Figures 5A and B). Perhaps nuclear fragmentation is the apoptotic landmark event indicating the irreversible stage of apoptosis.

### Reversibility of apoptosis in different inducers and in various cancer cell lines

**Figure 6:**
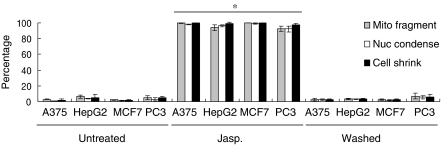
Reversibility of jasplakinolide-induced apoptosis in various cancer cell lines. Percentage of cells showing mitochondrial fragmentation (Mito. Fragment), nuclear condensation (Nuc. Condense), and cell shrinkage (Cell Shrink) of the control A375, HepG2, MCF7 and PC-3 cells (Untreated), the corresponding cells treated with 0.5 *μ*M jasplakinolide for 3 h (Jasp.), and washed and cultured with fresh medium for 24 h (Washed). (Mean±s.d.; ^*^P<0.02; *n*=3).

Our discovery on reversibility of jasplakinolide-induced apoptosis in HeLa cells evoked a novel phenomenon that cancer cells could survive after initiation of apoptosis, and therefore prompted us to investigate whether this was a general phenomenon in different apoptotic inductions and in various cancer cell lines. Our results on other apoptotic stimuli to HeLa cells showed that, in response to the induction of apoptotic inducers ethanol and staurosporine (Bertrand *et al*, 1994; Young *et al*, 2003), HeLa cells underwent apoptosis characterised by excessive mitochondrial fragmentation, nuclear condensation and cell shrinkage (Figure 4). After removal of the inducers, the indicated cells regained their normal morphology in 24 h in the culture of a fresh medium. In further experiments, jasplakinolide was applied to various cancer cell lines, which are widely used in cancer research including human skin cancer A375, liver cancer HepG2, breast cancer MCF7 and prostate cancer PC3 cells, and all of them displayed the morphological features of apoptosis (Figure 6). Consistently, after removal of jasplakinolide, the morphological recovery was observed in 24 h in all the cell lines (Figure 6). These results suggest that the reversibility of apoptosis is a general phenomenon in cancer cells.

## Discussion

In this study, we have shown that cancer cells could survive after initiation of apoptosis induced by different stimuli, and the reversibility of apoptosis was observed in various cancer cell lines. We provided evidence that cancer cells could escape from demise even after the cells undergoing critical apoptotic events such as mitochondrial fragmentation and dysfunction, nuclear condensation, cell shrinkage and activation of caspases. Importantly, the reversibility of apoptosis was abolished when the cells reached the apoptotic event of nuclear fragmentation, suggesting that this is an important cellular event indicating the point-of-no-return in apoptosis.

Our discovery on the reversibility of apoptosis in cancer cells lead to several unanswered key questions: what are the components of the machinery driving the reversibility of apoptosis, and how are they linked to the regulation of apoptosis in cancer cells as a whole? To what extent does the reversibility of apoptosis contribute to the survival and repopulation of cancer cells during the cycles of anticancer treatment? Intriguingly, can inhibition on the reversibility of apoptosis in cancer cells suppress cancer relapse? Providing answers to these questions will be critical in understanding the mechanism for regulation on the reversibility of apoptosis, and provide us new potential targets for therapeutical advancement to our war of cancer. A more in-depth analysis will be required to clarify these points.
