# Supplementary material for: Reversibility of apoptosis in cancer cells
Source: Br J Cancer. 2008 Dec 16;100(1):118–22. doi: 10.1038/sj.bjc.6604802 (PMC2634673; doi:10.1038/sj.bjc.6604802)
Supplement: Supplementary Information [file 6604802x2.doc]

**Supplementary Information**

Video 1 **Reversibility of apoptosis in HeLa cells**

Real time living cell imaging of the same HeLa cells before 0.5 µM jasplakinolide induction (Untreated), under the induction of the jasplakinolide (Induced), and after the cell washed and further incubated with fresh culture medium (Washed). Merged images: mitochondria (red) and nucleuses (blue) were visualized by fluorescence, and cell morphology was by DIC. Time presented as hr:min:sec.
